# Sensor Architecture and Task Classification for Agricultural Vehicles and Environments

**DOI:** 10.3390/s101211226

**Published:** 2010-12-08

**Authors:** Francisco Rovira-Más

**Affiliations:** Departamento de Ingeniería Rural y Agroalimentaria, Universidad Politécnica de Valencia, Camino de Vera s/n, 46022 Valencia, Spain; E-Mail: frovira@dmta.upv.es; Tel.: +34-963-877-291; Fax: +34-963-877-299

**Keywords:** sensor architecture, intelligent vehicles, off-road autonomous vehicles, robotics, precision agriculture

## Abstract

The long time wish of endowing agricultural vehicles with an increasing degree of autonomy is becoming a reality thanks to two crucial facts: the broad diffusion of global positioning satellite systems and the inexorable progress of computers and electronics. Agricultural vehicles are currently the only self-propelled ground machines commonly integrating commercial automatic navigation systems. Farm equipment manufacturers and satellite-based navigation system providers, in a joint effort, have pushed this technology to unprecedented heights; yet there are many unresolved issues and an unlimited potential still to uncover. The complexity inherent to intelligent vehicles is rooted in the selection and coordination of the optimum sensors, the computer reasoning techniques to process the acquired data, and the resulting control strategies for automatic actuators. The advantageous design of the network of onboard sensors is necessary for the future deployment of advanced agricultural vehicles. This article analyzes a variety of typical environments and situations encountered in agricultural fields, and proposes a sensor architecture especially adapted to cope with them. The strategy proposed groups sensors into four specific subsystems: global localization, feedback control and vehicle pose, non-visual monitoring, and local perception. The designed architecture responds to vital vehicle tasks classified within three layers devoted to safety, operative information, and automatic actuation. The success of this architecture, implemented and tested in various agricultural vehicles over the last decade, rests on its capacity to integrate redundancy and incorporate new technologies in a practical way.

## 1. Introduction

A United Nations panel held in 2008 concluded that “the way the world grows its food will have to change radically … to cope with growing population and climate change” [1]. The *Green Revolution* of the last fifty years, including efficient irrigation systems, productive varieties, and the high impact of mechanization to massively apply sophisticated pesticides and synthetic fertilizers has more than doubled traditional yields. However, these technological advances have come with ecological costs resulting in aquifer depletion, salinized soils, chemical poisoning from overuse of chemicals, and polluted streams. The fact that we have been consuming more food than farmers have been producing for most of the past decade led Bourne [1] to conclude that in order to meet rising food demand we need another green revolution, and we need it in half the time. Different alternatives have been proposed to achieve this *greener revolution*, such as biotechnology and sustainable farming, but the more immediate solutions brought by the so-called new technologies, *i.e.*, precision farming and agricultural robotics, seem to better match the revolution sought. The incorporation of these technologies into agricultural production not only benefits productivity and environmental conditions, but it also improves the working conditions of farm managers, laborers, and vehicle operators. In a study reported by Wexler [2], professionals whose work requires long hours alone in monotonous sensory environments have described alterations in mood, perception, and cognition resulting from extended periods of decreased sensory stimulation. However, many of the deficits associated with sensory deprivation can be prevented if the subjects exercise during the deprivation period. This is the situation encountered when tractor drivers spend most of their working time planting or spraying in monotonous fields, and even more severely when harvester operators employ day and night to have the yield collected on time when the moisture level is optimum, the price favorable, and weather conditions acceptable. The mental fatigue incurred by these drivers is not only caused by the monotony of the work, but also due to the stress created by the need of steering accurately within tight rows and lanes without causing any damage to the vegetation while maintaining a suitable working pace. The relief of the operator from driving continuously, very much like airline pilots, allows a multiplicity of actions that help to improve the realization of tasks while reducing the exhaustion of the driver. Furthermore, having more opportunities to interact with the external world, the additional information supplied by robotized vehicles lets operators make wiser data-based decisions. The proper integration of the set of sensors and automatisms that will make future vehicles more intelligent is key for the successful consummation of a new agricultural revolution.

The singular conditions found in agricultural production, especially the drudgery and repetitive nature of usual tasks, has motivated the wish for farm machinery automation since as early as 1924, when Willrodt [3] designed a steering attachment capable of following furrows to guide a machine automatically across the field. Until the appearance of electronics and computers, the sensing devices used to automate operations were purely mechanical. In fact, the majority of sensors used in agricultural vehicles have been related to autonomous navigation. For this purpose, the devices used both in North America [3] and in Europe [4] have been mechanical feelers, computer vision cameras, global positioning systems, geomagnetic direction sensors, laser scanners, and ultrasonic rangefinders. However, there are many other sensors of frequent use in precision agriculture such as yield monitoring estimators, soil properties probes, moisture content analyzers, and many others being developed at present. The usage of sensors in agricultural vehicles has evolved through time. In a study of patents devoted to in-field automatic navigation, Rovira-Más [5] found that beacons, pseudolite localization devices, and optical sensors excluding cameras were popular during the period 1985–2000, but inertial measurement units, GPS-based applications, and imaging devices became predominant in the 2001–2008 period. The particular case of GPS can be justified by the cancellation of selective availability in May 2000, which permitted the use of more accurate positioning data for civilian applications. The variety and use of sensors in agricultural vehicles has been increasing in the last two decades. Some of them, such as GPS receivers, have gained universal acceptance. Other sensors, on the contrary, coexist for the same purpose. Let us take, for instance, the case of feedback sensors for auto-steering control loops. Large auto-guided tractors typically insert flow meters in the steering mechanism, whereas smaller vehicles estimate the angle turned by the steering wheel with linear potentiometers or optical encoders affixed to the steering cylinder [6]. Most of the information managed within the framework of precision farming is expressed in terms of globally-referenced two-dimensional maps, as for example yield, soil, thermal, or variable rate prescription maps. This procedure requires a vehicle equipped with a global positioning receiver, normally a GPS, coupled with the specific sensors measuring the field properties represented in the maps. As technology advances, richer and more accurate information is being incorporated in the intelligent systems of vehicles, moving from two-dimensional maps to real time three-dimensional representations of agricultural scenes [7]. Far from being an exhausted topic, the selection, installation, combination, and actuation of sensors under a particular architecture onboard agricultural vehicles is in growing interest, not only for researchers, but for manufacturers and end-users alike. When we think about vehicle architecture, we can focus on embedded behaviors and the software capable of implementing them, we can center our attention on the hardware needs to sense and actuate according to those behaviors, or we can follow a holistic view and consider both. This article is primarily concerned with the second approach, that is, with the sensor architecture necessary to adapt conventional agricultural vehicles—no longer conventional—to farming tasks and field environments. The place and way to integrate behavioral and actuation modes will be discussed along the text. The set of behaviors that are susceptible to form the intelligent system of a vehicle is boundless, although there exist some of them which have gained broad acceptance of the robotics community. Blackmore *et al*. [8], for instance, provide a list of behaviors for an autonomous tractor, where simple processes as watching and waiting mingle with complex tasks such as route planning and navigation. The implementation of these behaviors on the tractor follows the work of Arkin by which the design of vehicle architecture is identified with software architecture rather than hardware, and consequently an object-oriented system architecture, very much like that used by software developers, is proposed to control the vehicle. While software failures have been identified as one of the major sources of problems for automating agricultural vehicles, hardware malfunction and disadvantageous sensor assemblages on vehicles have resulted in reliability issues as severe as, or even more than, software problems. For that reason, sensor architecture is assumed (in this paper) to be the first step in the design of the more general and complete vehicle architecture encompassing hardware, software, environments, target tasks, and vehicle dynamics. Yet, the design of the sensor network is heavily influenced by the rest, and therefore cannot be undertaken in isolation. The main objective of this article is to propose a sensor architecture that better adapts to the requirements and needs which make agricultural environments and tasks so special, and significantly distinct from other robotic applications. It is important, though, to always keep in mind that the degree of autonomy pursued hitherto with agricultural vehicles is *semi-autonomy*, which implies that an operator always remains in the cabin for security reasons. Full autonomy, however, will come in a further future if it ever comes.

## 2. Sensing Needs in Agricultural Environments

**Figure 1.**
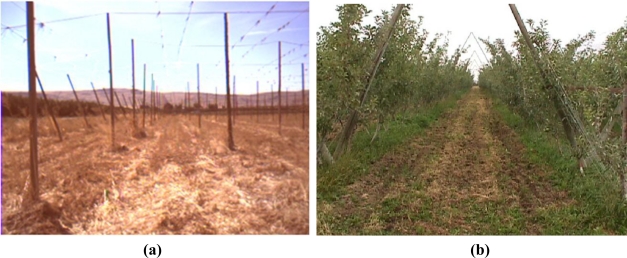
**(a)** Structure to support hops. **(b)** Trellis in apple trees.

**Figure 2.**
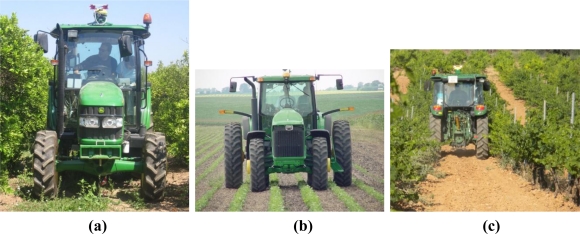
**(a)** Environment 3: citrus orchard. **(b)** Environment 4: soybean field. **(c)** Environment 5: Vineyard lanes.

There are three basic properties of agricultural systems that make these systems ideal for robotic applications: first, tasks are hard and very often exposed to harsh environments caused by extreme temperatures, strong sunlight, dust, or chemical spray; second, usual duties are repetitive, fatiguing, and time consuming; and third, the presence of crop rows, tree lanes, and supporting structures (Figure 1) offers a semi-structured setting from which intelligent vehicles can extract critical information. The vast majority of farming scenes taking place in open fields—*i.e.*, outdoors—can be classified under one of the following environments:

1. Barren fields ready to be ploughed, disinfested, or planted.
2. Swaths of cut crops prepared to be picked or baled.
3. Lanes delimited by rows of fruit trees whose height impedes normal vehicles to travel over them, as the citrus orchard of Figure 2(a).
4. Rows of bulk crops or short plants easily traversable in all or most of their growing time, as the soybean field shown in Figure 2(b).
5. Lanes of grapevines which can be traversed by tall vehicles but not by conventional tractors as illustrated in Figure 2(c).

All the environments described above, excluding the barren field, provide the perception system of intelligent vehicles with a number of detectable features that can be helpful to generate informative maps or navigate under automatic modes. However, plants, trees and supporting structures also limit the free space that vehicles need to carry out their regular tasks. As a matter of fact, the tight spacing left between crop rows or tree lanes usually poses complex challenges to the automation of agricultural tasks. The three examples of Figure 2 illustrate this fact; the allowable deviation available between row and tractor front wheels in Figure 2(b) is below decimeter levels, and there is not much room for steering corrections in the orchard of Figure 2(a) or the vineyard of Figure 2(c). The positioning accuracy demanded in such situations results in the highest level of precision required by GPS receivers, where sub-inch accuracies reached by Real-Time-Kinematic (RTK) GPS are becoming more and more popular among producers. Not other civilian application, not even aeronautics, is currently demanding such levels of accuracy. Nevertheless, not all the challenges come from high accuracy positioning needs; being aware of a vehicle’s surrounding is a fundamental capacity for an intelligent vehicle. A wide variety of unexpected obstacles may interfere with an operating vehicle: other farm vehicles (manually or automatically driven), forgotten tools, livestock, and even field laborers or hired personnel not always familiar with agricultural equipment. A satellite-based localization system will never account for mobile or unexpected obstacles, therefore there is a real need for an onboard perception system. Apart from detecting potential obstacles, local perception can provide small adjustments that help to place the vehicle in the optimum course. The perception system of an intelligent vehicle will be as complex as its sensing needs. If, for instance, people are being tracked or water stress in plants wants to be estimated, an infrared thermocamera will have to be installed. When nitrogen content or leaf disease is monitored, a hyperspectral camera will be added. A robotic shepherd might identify cattle or sheep with a monocular camera, but if tree density or three-dimensional mapping is pursued the choice will be made by selecting between a stereo camera and a laser rangefinder. In any case, regardless of the level of complexity accomplished, the operating mode of robotic off-road vehicles will be *semi-autonomy*, which always places an operator in the cabin for safety and reliability failures. The design of the sensor architecture has to consider this assumption, at least in the near and middle future. The fact that obstacles need to be detected by vehicles in certain applications does not imply that obstacles have to be unambiguously identified, which is a much more difficult task. For these situations, the sensing needs of the vehicle could be, for instance, determining whether an obstacle intercepting a programmed path is traversable or not, which can be graphically analyzed with *traversability* maps.

## 3. Special Vehicles for Specific Tasks

The particular configuration of agricultural vehicles, in comparison to other land vehicles, is the result of more than one hundred years of evolution since the dawn of mechanization. During this period of time, farm equipment has been adapting to the specific needs and requirements of field tasks. Given that most of these tasks have not changed profoundly, robotized vehicles will have to feature new capabilities without detriment of their previous ones. So, for instance, an automated harvester will have to keep its efficiency rates cutting crops and separating grain, and an autonomous sprayer will need to traverse off-road terrains at the stipulated velocities. The implications of this assumption are essential to understand and design intelligent agricultural vehicles, as they are totally different from small lightweight laboratory prototypes. Robotic platforms fulfilling farm duties need to withstand harsh atmospheric conditions, traverse rough terrains, possess working-day autonomy, deliver a minimum mechanical power, carry an operator (at least in the near future), keep up with current efficiency rates, and most importantly, assure high degrees of safety for all the operations involved. Typical agricultural vehicles weigh between 2 and 20 tons, incorporate diesel engines with a rated power between 20 kW and 500 kW, and can reach retail prices over $300,000. These figures bring the following advantages to automation and robotization of agricultural vehicles. First, the problem of heavy *payloads* for small robots; adding sensors and batteries to several-ton vehicles will have no effect on the vehicle’s stability and performance. Second, the *cost effect*: some electronics have a significant price which can represent an important percentage of the total cost of usual robots, but percentages quickly diminish when robotizing $70,000 tractors or $250,000 combines. And third, the typical problem of batteries and *autonomy*. Small robots can only carry a limited number of batteries, and in consequence cannot run for extended periods of time. Solar energy has been enough to power light planetary rovers but is insufficient to deal with regular power-demanding field duties. However, off-road vehicles powered by potent diesel engines can easily draw some hundreds of watts from the propulsion engine to energize all the electronics integrated in the vehicle. In addition, the largest part of present day agricultural vehicles feature a well conditioned cabin that is ideal to host electronics and delicate components in a protected environment.

The set of reasons adduced above are all favorable to vehicle automation. However, the possibility of an oversized, overweight, and extremely powerful machine getting out of control poses serious challenges to the commercial deployment of intelligent agricultural vehicles. In fact, this is the principal cause for delaying the general production of automated mobile equipment, as major manufacturers cannot assume this sort of risk. Moreover, a serious accident provoked by an autonomous machine would probably suspend research in the field for a long period of time. This issue takes us to the *paradox of vehicle size: reliability versus productivity*. A recurrent question in many robotics and automation conferences is whether future equipment will increase or decrease its size. Larger sizes make the control of automated machines critical; yet, minimum rates of productivity have to be granted. A trade-off will likely offer the best answer. In general, oversized vehicles might have their mass reduced but a minimum dimension, perhaps that of medium size tractors, may lead to a workable figure. Solutions, however, can follow two trends; the robotization of *conventional equipment*, and the creation of *innovative vehicles* endowed with specific capabilities. The former group is represented by usual tractors, harvesters, sprayers and so forth; and the latter includes automated utility vehicles, scouting robots, or site-specific flying applicators. Additionally, there has recently been a growing interest in the automation of golf vehicles and domestic gardening products such as lawnmowers, green mowers, and turf vehicles.

**Figure 3.**
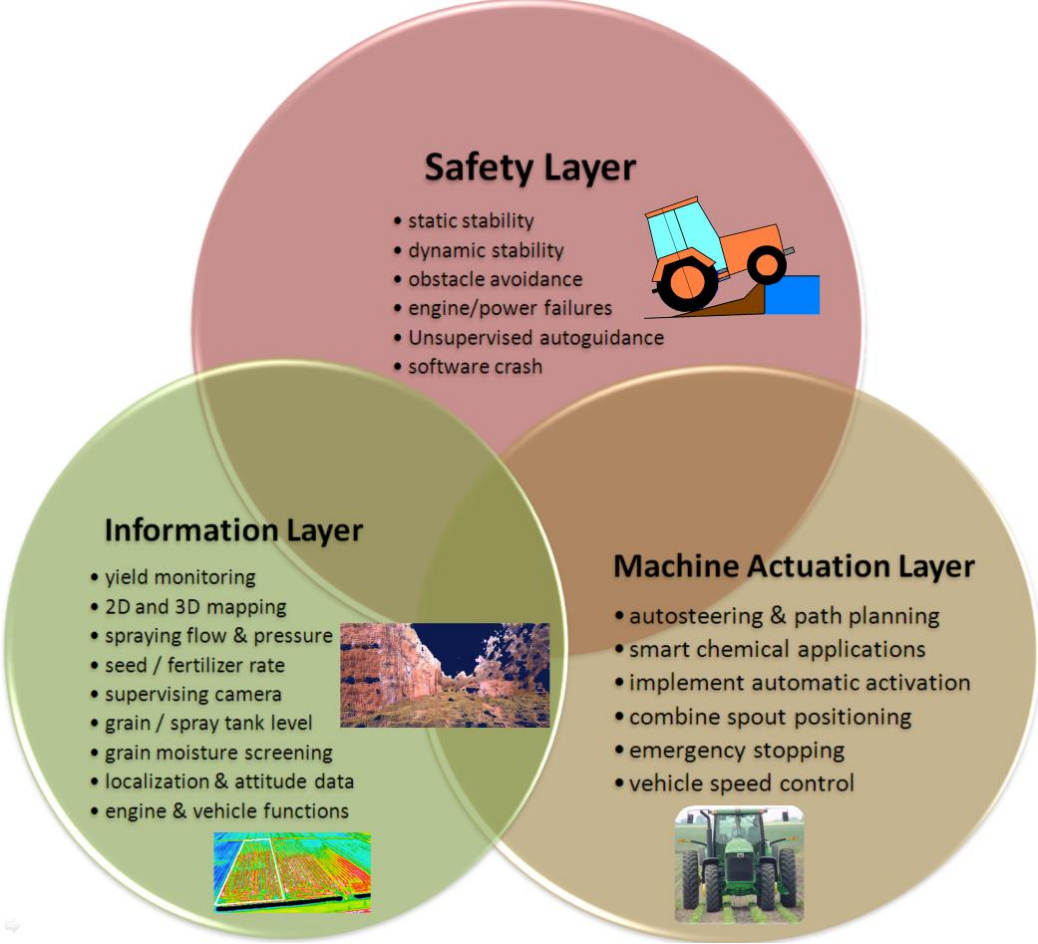
Three-layer task classification for intelligent agricultural vehicles.

The development of agricultural vehicles has been strongly influenced by the diversity of tasks and competences required in the field; therefore, it is convenient to consider the *binomial vehicle + task* when incorporating new capabilities, what means that vehicles not only have to adapt to the agricultural environments previously described in Section 2 but also to the duties commanded. The variety of tasks that can be automated is countless, from harvester spout adaptive positioning to grain truck convoy following. The addition of capabilities, and therefore of sensors, tends to be gradual according to producers’ needs and the maturity state of technology. The organization of the intelligent system embedded in the vehicle can be distributed into the three layers represented in Figure 3: Safety, Information, and Machine Actuation. The *Safety Layer* is the layer holding the highest priority, and is responsible for granting vehicle static and dynamic stability, path clearance and safeguarding, software and hardware reliability, engine proper behavior, and the safest use of automatic modes, preventing, for example, that the operator abandons the cabin while in automatic steering. In fact, some commercial auto-steering systems include a load cell under the driver seat, halting the vehicle as soon as the weight sensor detects the absence of the driver. California law, however, allows tractors in furrows traveling less than 3 km/h to travel without a driver, provided the throttle, clutch, and brakes can be controlled remotely, which implies a non-trivial state of automation. Interestingly, this practice is widespread with harvest crews [9]. Nevertheless, in spite of all the actions—both legal and technical—undertaken to reduce the number of accidents, agricultural machines are involved with many risky situations even before the implementation of automated systems. The United States Bureau of Labor Statistics indicates, for the year 2007, that the agricultural sector has the highest rate of occupational fatalities among high-rate sectors [10]. The evolution of agricultural equipment through the gradual incorporation of new technology offers a unique opportunity to improve the safety of field operations as well as the producers’ life standard. The *Information Layer* receives the inputs coming from the set of sensors onboard, and provides key information for decision making algorithms, storing historical data and fabricating prescription maps commonly used in precision farming applications. As a result, this layer is inherently passive as it gets information from the environment and distributes it throughout the other layers but it does not result in the activation of automatic actuators. Sometimes this information is processed in real time, for example when it takes part in safety warnings or steering assistance. On other occasions, the Information Layer supplies the producer with important knowledge for the management of the farm, such as yield spatial distribution, soil fertility, or water stress. Finally, the *Machine Actuation Layer* is in charge of executing orders and commands from reasoning algorithms and decision-making routines. These algorithms confer the vehicle with its *intelligent behavior* and continuously need to be fed by the onboard sensor network. This is, therefore, an active layer hosting from basic control loops to sophisticated behavioral-based architectures of the type outlined in [8]. Typical actions handled by this layer are variable rate planting, steering, implement positioning, smart spraying, or emergency braking. Although these three principal layers have been enunciated independently, it is obvious that they are deeply interrelated; safety requires both reliable information and accurate actuation, and every action exerted automatically needs precise feedback information from the onboard sensors to close control loops and grant stability. The Information Layer, on the other hand, may sometimes run independently from the other two layers when it basically provides managerial data to be stored and not processed in real time, as for example for the elaboration of yield monitoring maps or historical evolution graphs of soil fertility.

## 4. Results and Discussion: Sensor Architecture

**Figure 4.**
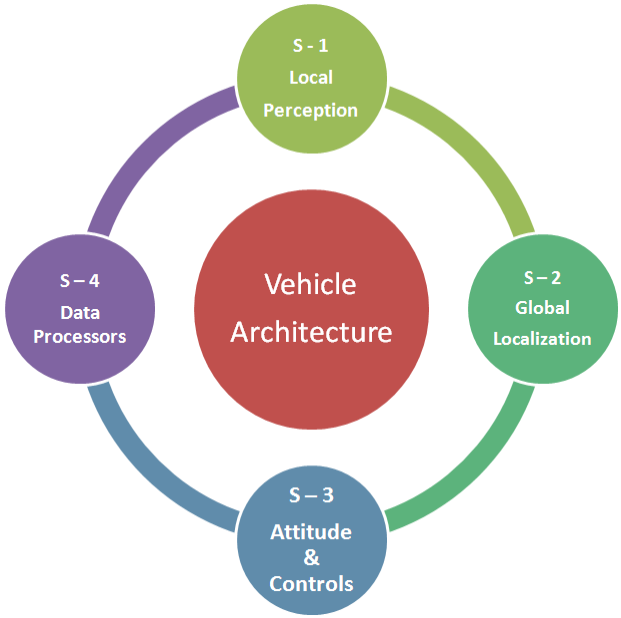
Four-core subsystem architecture for intelligent agricultural vehicles.

The *sensor architecture* proposed to meet the requirements of agricultural environments, vehicles, and tasks described in previous sections is in line with the general vehicle architecture presented in [11], and graphically represented by Figure 4, in which the entire intelligent system is articulated around *four structural subsystems*: local perception, global localization, actuation and control, and data processing. The fourth subsystem, *data processing*, comprises the set of computers, processing units, DSPs (digital signal processors), and embedded controllers hosting decision making algorithms, receiving sensor data, and sending actuation commands according to a given software architecture. The other three subsystems incorporate a multiplicity of sensors that have been grouped in the subsections 4.1 to 4.4 developed below. There will be, additionally, other ancillary components as extra batteries, power converters, signal conditioners, or emergency stop buttons, which assist the entire vehicle and therefore do not belong to any of the four structural subsystems illustrated in Figure 4. The practical necessity of redundancy and sensor fusion to enhance reliability forces the fluent cooperation among the four key subsystems, which is favored by the modular designed proposed. A popular example is given by the combination of local information captured by lidars or machine vision (perception subsystem) with global localization provided by a satellite navigation system. Two crucial aspects need to be considered before fusing the information coming from different sensors: *frequency* and *system of coordinates*. It is evident that global localization and (local) vehicle-fixed positioning will naturally have different coordinate systems, and therefore the appropriate coordinate transformation will have to be carried out before merging the data. Each sensor will generate readings at a particular rate, however there will be a main loop frequency at which the orders are executed by the processing subsystem. Main loop frequencies in the order of 10 Hz have been proved successful for operating off-road vehicles autonomously [12]. Imaging sensors can acquire images at 30 frames per second (fps), and inertial sensors can easily reach higher frequencies, but GPS receivers are typically set to send data strings at either 1 Hz or 5 Hz, which are below the recommended execution rates. Consequently, a suitable architectural design needs to be implemented in order to achieve the best performance for the selected onboard sensors.

### 4.1. Sensors for Local Perception and Vicinity Monitoring

The information that an intelligent vehicle can extract from its surroundings is much richer than that gathered by humans. Laser rangefinders can accurately trace profiles at distances where the human eye can only distinguish vague forms, ultrasonic devices make use of frequencies unnoticeable to human hearing, telephotos can see detail far away while macro lenses can perceive the detail of the microscopic. Near infrared, ultraviolet, or thermal infrared are bands of the light spectrum that are unreachable for us. High speed shutters can freeze rapid movements and vibrations. Human perception is greatly impaired at night but there are several sensors that work under scarce natural light. Yet, the human brain is unique and there is no computer comparable to it. For that reason, intelligent vehicles need to optimize their sensing capabilities in order to, somehow, compensate for the weaker reasoning capacity in comparison to human operators. The vicinity of an agricultural vehicle provides perception systems with critical information for vehicle mobility as well as on valuable data for improving productivity. Such endeavors as simultaneous localization and mapping (SLAM), crop-track guidance, three-dimensional (3D) terrain mapping, obstacle detection or avoidance, nitrogen content mapping, vegetation health monitoring, water stress early detection, and many other activities strongly rely and depend on the local perception subsystem.

*Ultrasonic* rangefinders have traditionally been well accepted for small robots roaming indoors, and under closed controlled environments, because of their affordability. A battery of several sonars affixed to the chassis of interior robots has been common to assist in navigation tasks. However, the larger size of agricultural vehicles and the needs of detecting complex environments in real time have limited their use due to the narrow field typically covered by ultrasonic devices, which in practice would result in an excessively dense network of sensors. A more convenient alternative to map ranges is offered by *lidar* (light detection and ranging) heads, optical devices based on the principle of time-of-flight whose beams of coherent light—usually laser—provide a way to estimate ranges with high resolution. The main disadvantage of lidars is the need to spin the beam in order to cover the widest possible area in front of the vehicle, typically between 180° and 270°, which requires a mechanism permanently in rotation. The speed of this circular movement limits the real-time capabilities of the sensor. Additionally, dusty atmospheres with substantial suspended matter can affect the precision of the range estimation. A double scanning platform rotating simultaneously in two perpendicular planes can generate three-dimensional maps with a single laser beam, but the synchronization of both rotational movements leads to complicated practical solutions when compared to other alternatives such as stereoscopic vision.

**Figure 5.**
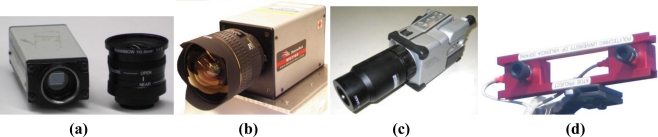
Main visual perception sensors for agricultural vehicles**: (a)** Monocular camera. **(b)** Multispectral camera. **(c)** Thermocamera. **(d)** Stereoscopic camera.

The great amount and diversity of information acquirable with vision sensors makes them indispensable in the general configuration of intelligent agricultural vehicles. The second “eyes of the operator” can actually see more than the operator as long as the right sensor, lens, and filter are mounted. The *monocular camera* of Figure 5(a) can work in the visible range and near infrared (NIR), and has been used to track crop rows and guide a tractor. When a NIR filter is mounted between the imager and the lens, only NIR reflectance passes through the filter, enhancing vegetation and facilitating the segmentation of the rows. With this kind of camera, the system integrator has to decide between visible spectrum or NIR band. The *multispectral camera* of Figure 5(b), on the contrary, grabs three images simultaneously in three predefined bands: red, green, and NIR. This option allows the combination of reflectance values from different wavelength intervals for exactly the same pixel areas, and therefore for the same features in the scene. It has been extensively applied to the monitoring of vegetation indices like the NDVI (normalized difference vegetation index). The displacement along the electromagnetic spectrum towards long-wavelength infrared intervals can be registered with *thermocameras* as the one shown in Figure 5(c). Thermographic maps have been used to detect water content in the field, water stress in plants, and as a safety feature to sense the presence of living beings immersed in tall crops. All the images mentioned so far and acquired with the cameras of Figures 5 (a,b,c) are two-dimensional images. When the three dimensions of space—X, Y, Z—need to be properly determined, monocular vision is not enough and *stereoscopic cameras* such as that of Figure 5(d) have to be incorporated in the perception system of the vehicle. Stereo vision is the perceptual system that best resembles human vision, where the pair of eyes is substituted by two identical cameras separated a horizontal distance denominated baseline.

**Figure 6.**
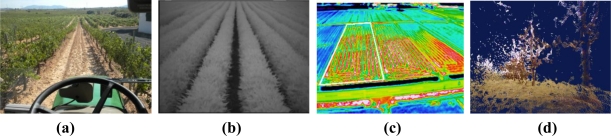
Typical images used in agricultural robotics**: (a)** RGB color image. **(b)** NIR-filtered monochrome image. **(c)** Thermographic map (Courtesy of N. Noguchi). **(d)** Stereoscopic image.

The availability of the three dimensions for every point—pixel—in the scene, habitually in the form of a 3D point cloud, provides a wealth of information every time an image is acquired. Since stereo cameras can estimate ranges, they have been used as safeguarding tools, and their outcomes are faster and richer than maps generated with lidars or sonars. Stereo-based 3D vision has also been used to automatically guide a harvester by detecting the edge of the crop being cut, and to recreate field scenes through virtual terrain maps [7]. The development of compact stereo cameras during the last decade has placed these sensors among the most cost-effective solutions commercially available. Camera manufacturers normally supply efficient correlation software to generate 3D clouds in real time. The main disadvantage of stereo cameras, however, is the high computational cost involved in 3D perception, especially when handling massive amounts of points, although the fast increase in processor speed given by Moore’s law is palliating this hindrance. Figure 5 shows four popular imaging sensors currently being used in agricultural robotics, and Figure 6 provides some sample images obtained with them. Figure 6(a) is a familiar RGB color image of grapevine rows acquired with a digital color camera. The field image of Figure 6 (b) has been altered with a NIR filter to highlight vegetation from soil and ease segmentation and thresholding. The thermographic map of Figure 6(c) correlates the temperature gradient of a field with the water accumulated in its soil. The virtual tree of Figure 6(d) corresponds to the 3D representation, in the form of a point cloud, of a tree surrounded by turf. Apart from the three Cartesian coordinates of every point, each pixel contains its RGB color code that helps to distinguish the detected trees from the surrounding yellowish grass.

### 4.2. Sensors for Global Localization

The generic name *Global Navigation Satellite Systems*, or GNSS, considers all the satellite-based global localization systems that can be publicly used for vehicle positioning. This technology was pioneered by NAVSTAR GPS (USA), and although the Russian independent system GLONASS has been updated with 20 operational satellites out of 26 in constellation (November 2010), the European Galileo is launching satellites, and the Chinese Beidou is under development, the only system fully operative worldwide at present is GPS. Nevertheless, a considerable effort is being made to assure compatibility among receivers such that a single receiver will be able to accept signals from a variety of global localization systems in the near future.

**Figure 7.**
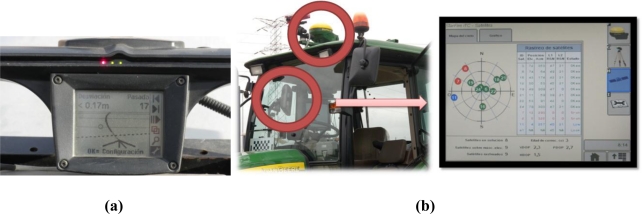
GPS for agricultural operations: **(a)** Lightbar steering assistance system. **(b)** DGPS onboard system.

The advent of GPS, especially reinforced by the suppression of the selective availability in 2000, has meant an extraordinary drive to the modernization of agricultural production, with the development of such revolutionary concepts as precision farming and agricultural robotics. The possibility of knowing the precise position of a vehicle in real time has opened the gold mine of information technology (IT) applications to agricultural fields; yield monitoring, variable rate prescriptions, and automatic steering all rely on GPS localization. The typical scenes portrayed in Figure 2 demonstrate the level of accuracy needed to navigate inside productive fields, where the space left for vehicle navigation is quite limited. However, not every operation requires the highest level of accuracy; automatic steering during harvesting demands the utmost precision, but yield monitoring or other mapping application with the purpose of creating historical maps can be successfully carried out with more modest equipment. Different alternatives are currently available for the average producer. The simplest one consists of a lightbar display that indicates how much the vehicle is offset from a predefined path by means of a horizontal set of lights, as shown in Figure 7(a). The driver, following the directions given by the lightbar, can follow a predefined course without the necessity of terrain marks. This procedure has been a cost-effective solution that became very popular at the beginning of the GPS era, and is still in use for some producers and common tasks. When the basic capabilities offered by multipurpose GPS manufacturers are not enough for a given field application, more sophisticated methods have to be implemented. Unlike airplanes and automobiles, off-road vehicles move around small areas where some important errors remain approximately constant. This fact may be used to correct the original signal received from the satellites with that emitted by a GPS reference receiver of well-known location, leading to the technique known as *Differential GPS* (DGPS). Satellite ephemeris and clock errors can be practically cancelled, but the mitigation of atmospheric errors degrades with distance [13]. Differential corrections improve localization data considerably, but not all sources of errors can be suppressed; multipath and receiver errors will still be possible. There are several ways to achieve differential corrections by establishing a network of reference stations distributed over moderate pieces of land (*Local-Area DGPS*) or over wide areas of the globe (*Wide-Area DGPS*). The latter has resulted in various specific systems according to the area of coverage: the North-American Wide Area Augmentation System (WAAS), the European Geostationary Navigation Overlay System (EGNOS), or the Japanese Multi-functional Satellite Augmentation System (MSAS). A wide-area DGPS can reach less than two meters positioning accuracy, but some agricultural operations require precisions at the decimeter level, which can be attained with commercial carrier phase differential signal providers. These private signal providers usually possess their own geostationary satellites to assure greater levels of accuracy, but users need to pay a periodic signal subscription. The top level of accuracy reachable with GPS is about two centimeters and can be accomplished with the *Real Time Kinematik GPS* (RTK-GPS). RTK sets contain two receivers, a radio link, and computer software with the purpose of enhancing GPS positioning accuracy by calculating differential corrections from a base station placed in the field, or nearby, where the vehicle is operating. The most important disadvantages of RTK systems are a coverage limitation of around 10 km between vehicle and base, and higher acquisition costs, although there is no need to pay additional subscription fees once the system has been implemented in the field. Figure 7 (b) shows a commercial DGPS installed in a tractor and some of the features displayed in the onboard user interface.

**Figure 8.**
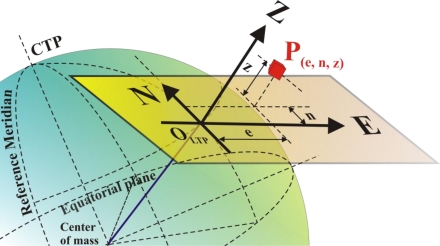
Local Tangent Plane system of coordinates for GPS agricultural applications.

Because GNSS technology is, after all, the result of satellite triangulation—in purity should be *trilateration* as ranges rather than angles are estimated—it can give the false impression that its applicability to the relatively small size of farms is somewhat low. The definition and adequate use of global systems of coordinates may account for this view. In fact, the conventional coordinate system in which GPS data is primarily output following the NMEA code format is the *World Geodetic System 1984* (WGS 84), an ellipsoid of revolution that models the shape of the earth. These coordinates—*latitude*, *longitude*, and *altitude*—seem to be inappropriate for the modest size of farms. However, given that the curvature of the earth has a negligible effect on agricultural fields, which can be considered flat in most of the cases, a more practical and intuitive system of coordinates can be used for agricultural applications; the *Local Tangent Plane* (LTP) system of reference. This reference frame allows user-defined origins close to particular operating fields, and employs the familiar orthogonal coordinates *North* (N), *East* (E), and *Altitude* (Z) as graphically defined in Figure 8. A step-by-step conversion between geodetic and LTP coordinates can be followed in [14].

### 4.3. Sensors for Vehicle Attitude and Motion Control

**Figure 9.**
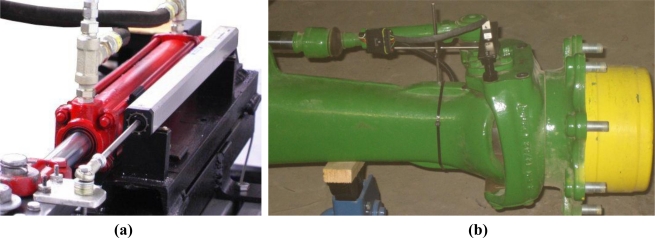
Wheel angle sensors: **(a)** Linear potentiometer. **(b)** Optical encoder.

The safe control of an agricultural machine is a complex endeavor, in which rich information collected from an uncontrollable environment needs to be quickly processed to assure a reliable and prompt response of the vehicle. Automated operations are regulated by feedback control systems whose execution loops include feedback sensors to track the variables under control. A basic need of intelligent vehicles is navigation assistance, which typically requires the real-time measurement of the angle turned by the wheels in front-axle/rear-axle steering, or the angular misalignment between front and rear bodies of articulated vehicles. The former case is especially relevant for off-road vehicles as many tractors, combines, sprayers, and self-propelled farm equipment in general achieve steering by actuating on the mechanical linkage that causes wheels to alter their orientation with respect to the chassis of the vehicle. The angle turned by front (front-axle steering) or rear wheels (real-axle steering) can be estimated with three sensors: linear potentiometers, flow meters, and optical encoders. *Linear potentiometers* [Figure 9(a)] give an indirect measure of the wheel angle by tracking the displacement of the cylinder rod actuating the steering mechanism. The calibration of potentiometers to correlate the voltage output by the sensor and the actual angle turned by the wheel is relatively simple. However, the assembly of a linear potentiometer on the steering linkage results too bulky sometimes, creating an easy trap for weeds and branches to get tangled in. A more compact solution, on the other hand, is available with oil *flow meters*. This alternative also implies an indirect measurement of the wheel angle by quantifying the oil flow moving in and out of the cylinder chambers to achieve a turn. While the interaction with branches and plants is practically inexistent because the sensor is internally integrated in the oil circuit, the need to manipulate and alter the primary fluid power circuit of the vehicle and the not always suitable accuracy of flow meters has reduced its universal use. The third option allows a direct measurement of the wheel angle with an *optical encoder*, a device comprising a free axle attached to a strapped disc whose position is easily tracked by a light beam. The ideal location for an optical encoder is right on the kingpin of the wheel, in such a way that the steering angle turned by the wheel is equivalent to the angle spun by the encoder axle. This solution requires that either the housing of the encoder or the axle affixed to the disc has to remain immobile when turning, what entails the design and assemblage of a customized frame in the usually constricted area close to the kingpin. Figure 9(b) illustrates this difficulty for the installation of two encoders on the kingpins of the front wheels of the medium-size tractor of Figure 2(a).

The dynamic analysis of a vehicle requires the estimation of the vehicle main states, such as position, velocity, acceleration, or the Euler angles roll, pitch, and yaw. These parameters are essential for applications involving autonomous operations, mainly if they include sensor fusion techniques like the Kalman filter. GNSS receivers can provide an estimate of the global position and average velocity of the vehicle, but the instantaneous attitude of the vehicle or its heading angle necessitates the complementary data given by inertial sensors. *Inertial measurement units* (IMU) combine accelerometers and gyroscopes, typically three of each disposed along the three orthogonal axes of a Cartesian frame. The accelerometers detect velocity changes over time—*i.e.*, the acceleration—and allow the calculation of speed and position by integration. The gyroscopes, on the contrary, are sensitive to instantaneous angular rates experienced by the vehicle around the main Cartesian axes. The integration over time of the three angular rates leads to the attitude angles yaw, pitch, and roll. Accurate IMUs tend to be costly, although their most notable disadvantage is the accumulation of error after extended periods of time, technically known as the sensor drift. The negative effects of drift need to be taken into account in agricultural environments where navigation paths tend to be fairly narrow. As a result, many navigation strategies incorporate sensor fusion methods to increase reliability. An inertial sensor is essential when the vehicle traverses terrains with significant slopes such as forestry exploitation sites, where roll and pitch are basic parameters for navigation and safety. However, the majority of agricultural fields are approximately flat, and therefore pitch and roll are usually negligible. In this situation, there are two vehicle states of great importance: heading and forward velocity. A straightforward means of estimating vehicle speed is by counting the number of rotations spun by the driven wheel with a *magnetic counter* mounted on the chassis. This calculation provides the theoretical speed of the vehicle, but due to the phenomenon of slippage, very frequent in off-toad terrains, the real speed of the vehicle does not usually coincide with the theoretical one, and therefore the theoretical cannot be used to estimate the actual speed. The theoretical velocity is useful, however, to calculate the vehicle slip as long as the real velocity is measurable with alternative sensors such as *radars*. The heading is a crucial parameter in the transformation from local to global coordinates, and in many path planning algorithms. It provides the orientation of the vehicle with respect to the north, and can be estimated with an inertial measurement unit as the yaw angle is determined by integrating the yaw rate around axes perpendicular to the local tangent plane. An optional sensor to estimate headings is the *fluxgate compass*, but the amount of electronic devices inside the cabin may create magnetic fields and affect the performance of the compass.

**Figure 10.**
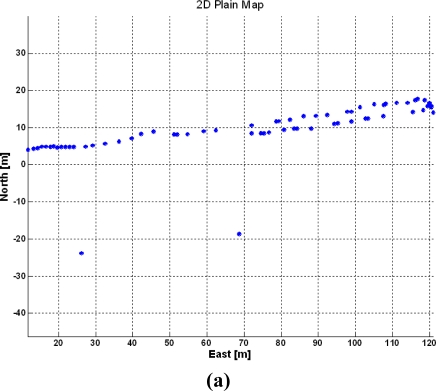

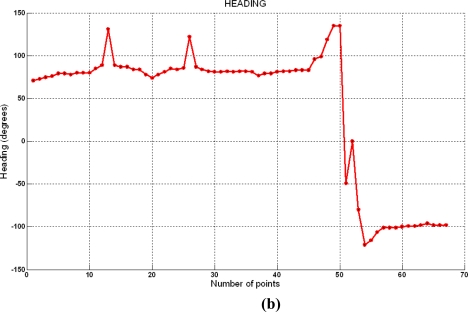
GPS-based heading estimation: **(a)** Vehicle trajectory. **(b)** Instant heading.

The fact that onboard GPS receivers can provide position and time for vehicles at a frequency of 5 Hz makes these sensors susceptible to estimate velocity and heading. This feature may or may not be acceptable according to the application pursued. To begin with, GPS heading cannot be known instantaneously unless a series of points have been properly recorded. But even in these circumstances, accuracy and signal stability have to be quite high; otherwise, fluctuations around average values will result in unacceptable headings, as instantaneous heading values will oscillate unrealistically. Additionally, the time needed to get stable series of data may be excessively long and no heading or speed will be available until the vehicle has traveled a significant portion of the planned course. The following two figures illustrate this issue when three-dimensional instantaneous mapping tests were conducted in a winery vineyard in the summer of 2010. Figure 10(a) shows the trajectory followed by the mapping vehicle [tractor in Figure 2 (c)] represented in local tangent plane coordinates; a complete row west-east, and half neighboring row in the return direction east-west. The tractor followed the straight lanes of the vineyard with approximate heading angles of +80° and −100° respectively, easily deducible from the GPS-based trajectory of Figure 10(a). The instantaneous heading for each point of the trajectory, estimated with an algorithm that considers 32-point series, is represented in Figure 10(b). At first sight, the plot seems stable and correct except for the two outliers noticeable in Figure 10, but when heading angles were introduced in the mapping algorithm designed to merge multiple 3D point clouds into a unique map, several inaccuracies showed up.

**Figure 11.**
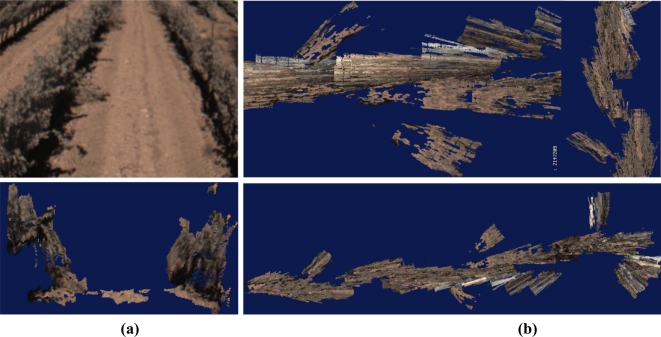
3D terrain mapping: **(a)** Sample image (top) with its 3D view (bottom). **(b)** Heading errors in top view.

Figure 11(a, top) provides one of the 67 RGB images of the vineyard lane used to construct the 3D map, and Figure 11 (a, bottom) displays its 3D view. The virtual representation of the vines utilizes true color to distinguish vegetation (dark green) from soil (light brown). A top view of the two virtual rows is given in Figure 11(b).

The individual maps with origins located at the coordinates given by the two outliers found in Figure 10 were automatically eliminated by the mapping algorithm and therefore do not appear in Figure 11(b), but they were the cause of large errors in the alignment of the images. Notice that many of these 3D images were correctly displayed, but when they were fused to complete the global map of the two rows, the lack of accuracy in the estimation of the heading resulted in frequent misalignments and defective orientation for various portions of the lane. The complete map is rendered in the bird-eye view of Figure 11(b, bottom), and two augmented portions are displayed in the top image. For this application, a more reliable source of headings is therefore necessary. Furthermore, the reliability of GPS was not satisfactory either, as two important outliers appeared, even though there were always between six and nine satellites in solution.

### 4.4. Non-visual Sensors for Monitoring Production Parameters

Apart from all the information acquired through the vision sensors mentioned in Section 4.1, there are other parameters that are important to producers and cannot be determined remotely. The real time estimation of the harvested crop is normally tracked by a *yield sensor* mounted inside the combine harvester. Average values of yield are globally referenced with a GPS receiver so that yield maps can be generated at the end of the season. Yield monitoring is popular for grain production, mainly corn and soybeans, as well as for wine production. Other interesting maps like those representing rainfall or soil properties cannot be built “on the fly”, generally speaking, as penetrometers, PH-meters, conductivity probes, and other sensors have not been incorporated to vehicles with normality yet.

The complete automation of an agricultural vehicle involves many more functions than automatic steering. Navigation, for example, may require gear shifting, brake activation, throttle control, or differential locking. All these actions, when executed automatically, need to track the position of levers and pedals with *potentiometers* and *encoders*. An intelligent implement, for instance, needs to sense its position (up for road traveling and headlands; down for farming) as well as the drag force incurred by the pulling vehicle (*axle load cells*).

### 4.5. Onboard Integration of the Complete Sensor Network

All the sensors that comprise the architecture proposed need to be optimally integrated in the intelligent agricultural vehicle for its use to be easy, comfortable, and safe. The physical location of the sensors is decisive and needs to be carefully planned. The main processor(s) of the vehicle, as well as monitors and screens, will preferably be installed inside the cabin, where vibration, dust, and moisture will have a minimum impact. Consequently, provisions should be made for setting a neat framework of multiple cables entering and exiting the cabin. A second battery, independent from the vehicle’s own battery, is always very helpful to preserve the desired autonomy of the diesel engine. Code debugging and the simultaneity of multiple sensors can easily exhaust the main battery when the engine is not running but computers and sensors are on. In addition, starting the engine results in temporary voltage drops that turn the onboard sensors off, invalidating previous initialization routines. GPS receivers, for example, need several minutes to lock the proper number of satellites, and every time the voltage is cut off, the constellation search needs to start over again. For this reason, an automated double-battery charge system is very convenient. This system for powering the added electronic devices was successfully implemented in the tractor of Figure 2(a,c), and it charges both batteries with the engine alternator when the engine is running, but powers all the electronics onboard just with the secondary battery. Only in the unlikely event of running the secondary battery out while the engine is off, the principal battery would power sensors and computers.

**Figure 12.**
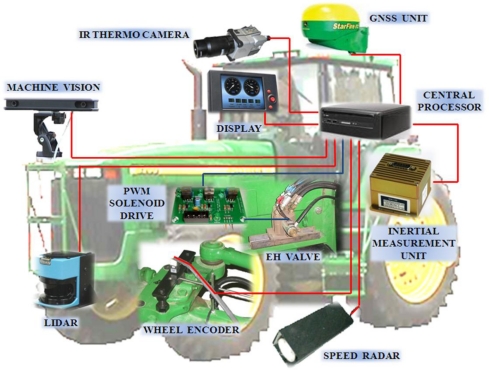
Generic sensor network for an intelligent agricultural vehicle.

The sensors which do not require a special position in the vehicle, such as compasses or inertial measurement units, are better kept in the cabin; they are well protected and connections—power and signal—are kept short. For many sensors, however, there is an advantageous, or even unique, location in the vehicle. Optical encoders, for instance, need to be mounted on the (front-axle) wheel kingpins, and therefore no other placement makes sense to directly track steering angles. The GNSS antenna receives better data when located high, as multipath reflections from the ground and from low vegetation can be avoided; thus, a centered position on the cabin roof is usually the preferred option. Lidars and cameras may be mounted either at the front of the vehicle or on the cabin, depending on the sort of scenes being sensed. The complexity of orchestrating all the sensors, actuators, and computers, while assuring the right voltage power and the synchronization of data acquired at various frequencies, calls for a well designed sensor and system architecture. Figure 12 shows a pictorial representation of a generic sensor network for an intelligent agricultural vehicle.

After the network of sensors onboard has been properly designed, choosing the optimum sensors for each subsystem, selecting their most favorable location within the vehicle, and linking them reliably with the main processor, it is time to revisit the three-layer task classification and discuss on the intelligent capabilities of the architecture proposed. There is no physical embodiment of the task layers because they are conceptually conceived as containers of such virtual elements as information, risk prediction, or expert systems. The *Machine Actuation Layer* is the layer that holds the set of algorithms conferring intelligence to the system, which may be physically located in the main computer, in several DSPs, or even in a multiplicity of sensor-based agents as the processing board of a smart camera. The design and interrelation of all these algorithms is what some authors consider to be the system architecture, although in reality it is the *software architecture*. The model envisioned in this article considers the *system architecture* to be the envelope that comprises both hardware and software architectures. The former refers to the sensor and complementary *hardware* described along this article. A detailed exposition of the latter would require another paper in the line followed by [8], although a generic view may be outlined here. Taken as a whole, the actuation plan for the vehicle can follow the biology-based reactive approach of the *subsumption architecture* developed by Brooks [15], or on the contrary it may include a *cognitive engine* inside the Actuation Layer. While both have been proved to perform successfully for a number of robots in particular situations, agricultural vehicles usually benefit from both approaches, and consequently the best results are often achieved with a hybrid model implementing ideas taken from both. Several software architectures for agricultural off-road vehicles are described in the study cases presented in [14].

## 5. Conclusions

The Aral Sea in Asia and Lake Chad in Africa have suffered a reduction of their surface down to 10% of their original size in barely 30 years, as a consequence of unwise decisions made by the agricultural sector [16]. The impact of the imprudent exploitation of natural resources affects large areas of the globe, often involving several nations. Because the way the world grows its food needs to change in order to fight famine while assuring sustainability, a technological revolution is called for. The application of new technologies to agriculture, through the disciplines of *precision farming* and *agricultural robotics*, can bring practical solutions to make food production more rational and efficient. Agricultural vehicles are privileged agents in which new technologies are currently being implemented. New ways of carrying out traditional tasks, such as automatic harvesting, variable rate applications, or water stress site-specific detection can be key in the future to assure novel production systems compatible with population growth and environment preservation. These technologies, however, require the optimum implementation of sensors, actuators, and computers in the so-called *intelligent vehicles*. This article proposes a *sensor architecture* to endow agricultural vehicles with the necessary capabilities to perform tasks within the framework of precision agriculture and field robotics. This sensor architecture, in conjunction with the software architecture, constitutes the vehicle system architecture. The hardware architecture developed defines four key groups, or families, of sensors: local perception and vicinity monitoring, global positioning, attitude and control, and non-visual tracking of production parameters. These four sensor families are normally present in most intelligent vehicles, although the particular sensors actually included in each group, depend on each specific application. The arrangement of sensors according to this architecture has favored redundancy and the practical implementation of new technologies in agricultural off-road vehicles, complying with especial requirements of tasks and environments. Table 1 provides a cross-table relating some advanced vehicle tasks with the sensors needed to accomplish them. Future needs will likely result in new tasks, novel sensors, and as a result an augmentation of the original architecture proposed; but the adoption of new technologies by the agricultural sector is a matter of time.

## Figures and Tables

**Table 1.**
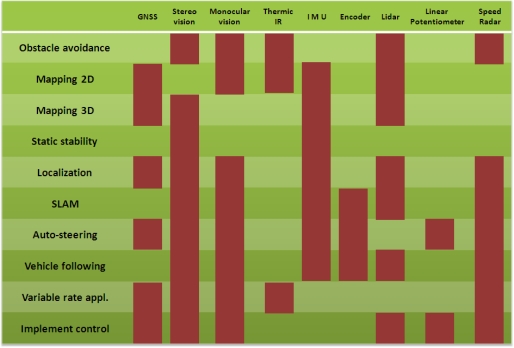
Sensor-task cross table.
